# The New Building of the Dental Department of the University of Maryland

**Published:** 1884-05

**Authors:** 


THE NEW BUILDING OF THE DENTAL DEPARTMENT OF THE UNIVERSITY OF MARYLAND.—Owing to the large classes, it has been found necessary to increase the size of the Dental Infirmary and Laboratory Building, by the erection of two extensive wings, which afford so much additional space to the large Infirmary Hall, as to give some *two hundred* running feet of continuous window fronts on the second story, and additional Museum, Reception, Impression and Extracting Rooms on the first story.

The Dental Laboratory now presents *one hundred and eighty* running feet of students' work tables. For eligibility of site and arrangement—having been erected especially for instruction in practical dentistry—this Dental Infirmary and Laboratory Building is not equalled by any other similar structure in this country. It commands unobstructed light from every side by means of *sixty-five* windows. The practice is very large, as frequently over twenty patients are in waiting in addition to those undergoing operations. The number of patients for the extraction of teeth is simply immense, the average number per day for each student reaching as high as *twenty*. The Infirmary practice is increasing to such a degree that all of the students now in attendance upon the Summer Practical Course have more than they can possibly attend to. No other dental school has superior facilities for obtaining patients, upon which students gain that practical experience so necessary in the practice of dentistry.

